# Clinical and molecular characteristics of fructose 1, 6 bisphosphatase deficiency in 6 Egyptian patients and two common variants

**DOI:** 10.1186/s13023-025-04100-9

**Published:** 2025-11-20

**Authors:** Solaf M. Elsayed, Radwa G. Mahmoud, Yasmeen Abdelaziz Fereig

**Affiliations:** 1 Department of Medical Genetics, Faculty of Medicine, Ain Shams University, Cairo, Egypt; 2 Department of Pediatrics, Faculty of Medicine, Ain Shams University, Cairo, Egypt

**Keywords:** Fructose 1, 6 bisphosphatase deficiency, *FBP1* gene, Hypoglycemia, Ketosis, Metabolic acidosis

## Abstract

**Background:**

Fructose 1, 6 bisphosphatase (FBPase) deficiency is a rare autosomal recessive disease caused by mutations in the *FBP1* gene. Symptoms of this disease are heterogeneous, with a variable age of onset, and are often confused with those of other inborn errors of metabolism. Biochemical testing is not conclusive, and patients usually need molecular testing for proper diagnosis and management.

**Aim of study:**

To describe clinical and molecular characteristics of patients with FBPase deficiency.

**Patients and methods:**

The study included six female patients diagnosed with FBPase deficiency, all recruited from the outpatient genetics clinic and the Children’s Hospital at Ain Shams University, Faculty of Medicine. The mean age at presentation was 22.8 ± 16.16 months, while the mean age at diagnosis was 62 ± 45.16 months, indicating an average diagnostic delay of three years. The most common presenting symptoms were vomiting, fever, and lethargy. Hepatomegaly was the most frequently observed clinical sign on examination. Initial laboratory investigations commonly revealed ketotic hypoglycemia and metabolic acidosis. Molecular testing confirmed the diagnosis in all cases. Encouragingly, with appropriate management, all patients achieved normal neurocognitive outcomes.

**Conclusion:**

Fructose 1,6-bisphosphatase deficiency should be considered in children presenting with hypoglycemia and metabolic acidosis. Early molecular diagnosis is recommended to confirm the condition and to facilitate carrier screening and preventive strategies for at-risk family members.

## Introduction

**Fructose-1**,**6-bisphosphatase (FBPase)** is a key regulatory enzyme in gluconeogenesis, catalyzing the hydrolysis of fructose-1,6-bisphosphate into fructose-6-phosphate and inorganic phosphate [1]. In 1970, Baker and Winegrad first reported FBPase deficiency in a child who presented with fasting hypoglycemia and metabolic acidosis triggered by fructose and glycerol. The condition was distinguished from hereditary fructose intolerance and glycogen storage diseases. FBPase deficiency is a rare metabolic disorder, with an incidence estimated between 1 in 350,000 and 1 in 900,000 in the European population [2]. Although there are no published data on its incidence in the Egyptian population, it is likely to be higher due to the increased prevalence of consanguineous marriages [3]. Clinically, the disorder presents with a heterogeneous spectrum, but the characteristic triad of hypoglycemia, ketosis, and metabolic acidosis is typically observed. Acute episodes are commonly precipitated by fasting, infections, or ingestion of a large fructose load [4–6].

Here, we describe the clinical and molecular characteristics of six Egyptian patients from unrelated families diagnosed with FBPase deficiency.

## Patients and methods

Clinical and laboratory data were collected retrospectively. All patients underwent molecular genetic testing using next-generation sequencing (NGS) with a gene panel targeting inborn errors of metabolism. The study was approved by the Ethical Committee of Ain Shams University (FWA 000017585), and informed consent was obtained from the legal guardians after they were fully informed about the study.

### Case presentation

#### Case (1)

The first patient was a 4-year-old girl, the fourth child of second-cousin parents. She presented at 2 years of age with fever, refusal of oral intake, and severe emesis and hematemesis. Similar presentations recurred, usually triggered by viral infections, but occasionally there was no precipitation factor. These episodes were preceded by thirst and lethargy. Initial investigations revealed metabolic acidosis, a mild increase in liver enzymes, and an increase in serum lactate. An extended metabolic screen (EMS), including amino acids and acylcarnitine, was done by Tandem Mass Spectrometry (TMS) revealed no abnormalities. A mitochondrial hepatopathy was suggested, and therefore, the patient received a mitochondrial cocktail plus maintenance oral sodium bicarbonate solution for persistent acidosis. With no definitive diagnosis, the patient was then referred to the genetics clinic at the age of 3 years. Her weight was 16 kg (0.81 SD) and height was 96.5 cm (0.08 SD). Abdominal examination revealed mild hepatomegaly, 4 cm below the costal margin, with a firm, rounded border and a smooth surface. Her spleen was not palpable. Chest and heart examination were normal.

#### Case (2)

The second case was a 3-year-old female, the third born to first-cousin parents. Her first episode was at the age of 14 months with fever, lethargy, and tachypnea. Primary investigations revealed metabolic acidosis, a mild increase in serum ammonia, and lactate. This episode recurred with a viral infection causing fever, cough, and recurrent vomiting. Investigations showed metabolic acidosis, ketotic hypoglycemia, and a mild increase in liver enzymes. EMS was normal. Differential diagnosis, including septic shock, mitochondrial hepatopathy, and liver failure, was excluded. The patient was then referred to the genetics clinic at the age of 28 months. Her weight was 10 kg (-1.87 SD), height was 84 cm (-1.55 SD). Abdominal examination revealed no hepatomegaly; The rest of the physical examination was normal.

#### Case (3)

The third case was a 5-year-old female, the third born to non-consanguineous parents. She presented at the age of 10 months with fever, impaired consciousness, metabolic acidosis, and hypoglycemia. At the age of 2 years, the episode was repeated with vomiting, convulsions, severe hypoglycemia (serum glucose was reported to be 15 mg/dl), and metabolic acidosis. Urine ketone test was + 2, serum ammonia and lactate were mildly increased, while EMS and urine organic acids were normal. Differential diagnosis, including viral or immune encephalitis, was considered, yet CSF results were negative. The patient was then referred to the genetics clinic at the age of 3 years and 4 months. Her weight was 15.8 kg (-1.39 SD), height was 102.5 cm (-1.99 SD). Abdominal examination was notable for mild hepatomegaly. The rest of the physical examination was normal.

#### Case(4)

The fourth case was a 4-year-old female, the second born to first-cousin parents (one of twins), who presented to the emergency department with impaired consciousness. Initial investigations revealed metabolic acidosis, hypoglycemia, and mild elevations in serum ammonia and lactate, while the EMS (extended metabolic screen) result was normal. The patient was subsequently referred to the endocrinology department. Her weight was 13 kg (-1.67 SD), and her height was 99 cm (-1.16 SD). An extensive workup for hypoglycemia, including a critical sample, failed to identify the underlying cause. Laboratory investigations excluded an endocrinopathy. Physical examination revealed mild hepatomegaly, with the remainder of the examination being unremarkable.

#### Case 5

The fifth case was a 10-year-old female, the second born to first-cousin parents, who presented at the age of one year with hypoglycemic convulsions and coma. This episode was repeated several times with metabolic acidosis, ketonuria, and mild hyperammonemia. EMS and organic acids in urine were normal. The patient was diagnosed with mitochondrial cytopathy and was maintained on a mitochondrial cocktail. On examination, her weight was 30 kg (-0.84 SD), height was 142 cm (-0.16 SD. Abdominal examination was notable for mild hepatomegaly; the rest of the physical examination was normal. Family history revealed a deceased brother with hepatomegaly and liver failure, and no available investigations.

#### Case 6

The sixth case was a 13-year-old female, born fifth to non-consanguineous parents. She was presented with episodic vomiting, hypoglycemia, and metabolic acidosis at the age of 2.5 years. She was referred to the genetics clinic at the age of 11 years. On examination, her weight was 78 kg (2.13 SD), her height was 170 cm (3.7SD). Chest, heart, and abdominal examination were normal. EMS was normal. Lactate was elevated. Organic acid in urine was done as a part of IEM workup, and it revealed elevated lactate, pyruvate, highly elevated ketones, 2-hydroxybutyric acid, and 2-hydroxyisovaleric acid. Pelvic abdominal ultrasound and Echocardiography were normal.

Tables 1 and 2 summarize the demographic and clinical findings of the six cases.

**Table 1:** Patients’ demographic data

Name	Age (year)	Sex	Consanguinity	Weight	SD (Wt.)	Height	SD(Ht.)
Case 1	4.5	Female	second cousin	16 kg	-0.68	96.5 cm	-2.31
Case 2	3	Female	First cousin	10 kg	-2.89	84 cm	-3.04
Case 3	5	Female	Non-consanguineous	15.8 kg	-1.39	102.cm	-1.99
Case 4	4	Female	First cousin	13 kg	-1.67	99 cm	-1.
Case 5	10	Female	First cousin	30 kg	-0.84	142 cm	-0.16
Case6	13	Female	Non consanguineous	78 kg	2.13	170 cm	3.7

**Table 2:** Patients’ clinical presentation

No.	Age at presentation (months)	Presenting symptoms	Mental state	Hypoglycaemia	Metabolic acidosis	Hepatomegaly	Age of molecular diagnosis (months)	Outcome
1	24	Fever, vomiting, hematemesis	Lethargic	Positive	Positive	Negative	36	Normal cognition, short stature
2	14	Fever, vomiting	Lethargic	Positive	Positive	Positive	24	Normal cognition, short stature
3	10	Fever, vomiting	Lethargic	Positive	Positive	Positive	36	Normal cognition and growth
4	48	Fever	Lethargic	Positive	Positive	Positive	48	Normal cognition and growth
5	12	Convulsions	Coma	Positive	Positive	Negative	84	Normal cognition and growth
6	30	Vomiting	Lethargic	Positive	Positive	Negative	144	Normal cognition and growth

Molecular testing was done on all cases using NGS for a gene panel of IEM. (Table 3)

**Table 3:** *FBP1* variants found in the six patients

Patient	Transcriptional Position (NM_000507.3)	Amino Acid Position	Mutation Type	Variant pathogenicity	Zygosity
1	c.469G > C	p. Gly157Arg	Missense	Uncertain significance	Homozygous
2	c.902_904del	p. Glu301del	In-frame deletion	Likely pathogenic	Homozygous
3	c.902_904del	p. Glu301del	In-frame deletion	Likely pathogenic	Homozygous
4	c.155 C > T	p. Ala52Val	Missense	Uncertain significance	Homozygous
5	c.469G > C	p. Gly157Arg	Missense	Uncertain significance	Homozygous
6			Deletion of Exon 1	Likely pathogenic	Homozygous

## Results

The patients’ ages ranged from 3 to 13 years, and all six individuals were female. The mean age at symptom onset was 22.8 ± 16.16 months, while the average age at diagnosis was 62 ± 45.16 months. This reflects a mean diagnostic delay of 39 ± 44.66 months. Molecular testing confirmed the diagnosis of FBPase deficiency in all cases, with two common variants in six different families.

### Treatment and follow-up

All patients are currently following a fructose-free diet. They have been advised to avoid prolonged fasting and to consume a meal containing uncooked cornstarch before extended periods of sleep, particularly in younger children, to help prevent metabolic crises—an approach consistent with other studies. Additionally, each patient was provided with an emergency letter to present at the emergency department during acute episodes.

## Discussion

This study presents the first case series describing the clinical and molecular features of fructose-1,6-bisphosphatase (FBPase) deficiency in Egypt. Our findings emphasize the diagnostic challenges posed by the clinical overlap with other IEM, which often result in delayed or incorrect diagnoses. Despite this overlap, the presence of the characteristic triad—hypoglycemia, ketosis, and metabolic acidosis—should raise clinical suspicion for this treatable disorder.

Elevated lactate level and recurrent hypoglycemia initially led to a misdiagnosis of mitochondrial cytopathy in several cases, a common diagnostic pitfall that was also noted in previous studies [7–10]. EMS (extended metabolic screen) and organic acid results were of no help in diagnosis, except in patient 6, where high glycerol was consistent with prior reports. Nevertheless, it is still non-specific except if the urine sample is collected in a period of metabolic crisis [8, 11]. These limitations underscore the importance of molecular genetic testing, particularly in the context of overlapping IEM phenotypes. Given the heterogeneity and non-specificity of biochemical findings, NGS is the preferred approach for definitive diagnosis.

Since the first mutation associated with FBPase deficiency was discovered, many pathogenic variants have been reported from different populations, with some reports from Arab countries, including Syria and Saudi Arabia [12–14], while this is the first report of genetically confirmed cases in Egyptian patients. Notably, two common variants were identified among six unrelated families, suggesting a higher-than-expected frequency of this disorder. Interestingly, only 66.7% of patients were born to consanguineous parents, hinting at a potentially higher carrier rate in the population.

The 469G >C (p. Gly157Arg) homozygous variant was present in both cases 1 and 5. Both patients had an early age of onset (between 1 and 2 years of age) with no hepatomegaly. The second variant was the homozygous in-frame deletion, c.902_904del (p. Glu301del), found in cases 2 and 3. Both had an early onset of symptoms (between 10 and 14 months of age) and presented with hepatomegaly. This same variant was previously reported in a patient with hepatomegaly [15].

Homozygous deletion of exon 1 was found in case six in this cohort. The same variant was reported in a recent study in Syria and was suggested as a founder mutation [16]. Exon 2 deletion, previously referred to as exon 1 has been reported as the most frequent mutation in Turkish patients [17].

Most patients in this series developed symptoms before the age of two, with a mean onset of 22.8 ± 16.16 months. However, neonatal-onset cases have been described in Turkish and Chinese populations as early as days 1 to 3 of life [18].

Hypoglycemic episodes often become less frequent with age, improving after early childhood, as noted in this cohort. In Case 6, symptomatic episodes significantly decreased after the age of eleven. Despite the improvement in symptoms over time, diagnostic delays were common, with a mean delay of 39 ± 44.66 months, and particularly late diagnosis in Case 6, consistent with observations in Turkish cohorts [19].

Fever was a common trigger for metabolic decompensation, aligning with previous literature [7]. Episodic elevations in liver enzymes were seen in cases 1 and 2 and have also been noted in earlier studies [13, 15, 16, 18–20]. Hypoglycemia was occasionally severe enough to cause convulsions and coma, a finding that echoes earlier reports [18].

## Conclusion

FBPase deficiency remains underdiagnosed due to its similarity to other disorders of energy metabolism. Early recognition of the clinical triad and the application of molecular diagnostics are crucial to improving patient outcomes. In our series, genetic testing will not only confirm the diagnosis but also facilitate carrier detection and offer reproductive options and family counseling, especially with a tendency of consanguineous marriage, Given the discovery of shared variants in unrelated families, further population-based screening studies are warranted to better understand the prevalence and carrier frequency of this condition in Egypt and the broader Middle Eastern region.

## Data Availability

Data is available upon direct contact with the authors.
